# 

**Published:** 1897-12

**Authors:** 


					﻿CONTENTS.
ORIGINAL ARTICLES:
Tenotomy in Convergent Squint.....................  583
Strangulated Hernia (Abstract)..................... 585
SELECTIONS AND ABSTRACTS:
The Dangers of Treating Gonorrhœa With Simple Astringents.	593
Surgical Uses of Picric Acid.......•............    595
The Wonders of Modern Medicine and Surgery......... 566
The Treatment of Cerebral Hemorrhage............... 606
The Treatment of Syphilis.......................    610
Inegular Menstruation in Young Women Due to Anemic Conditions 611
A New Test for Typhoid Fever........................614
SELECTIONS AND ABSTRACTS:
A Simple Method for Removing Foreign Bodies from the Nasal
Cavities of Children ..............................  615
Ligation of the Dorsal Vein of the Penis for Impotency . 616
Modern Technique of the Caesarian section............ 618
Iron and Manganese Peptonate......................... 620
Treatment for Insect Bites .... ................*.....	621
The Dietetic Treatment of Diabetes...................... 622
Specialists and Specialties ...........................  623
Venomous Snakes—A Request .......................   ,.	623
BOOK REVIEWS............................•................ 625
EDITORIAL.............................................    627
BUSINESS NOTES .........................................  631
PRESCRIPTION DEPARTMENT ...._	   633
				

